# Investigation of wound healing process guided by nano-scale topographic patterns integrated within a microfluidic system

**DOI:** 10.1371/journal.pone.0201418

**Published:** 2018-07-26

**Authors:** Insu Lee, Daegyu Kim, Ga-Lahm Park, Tae-Joon Jeon, Sun Min Kim

**Affiliations:** 1 Department of Mechanical Engineering, Inha University, Incheon, Republic of Korea; 2 Department of Biological Engineering, Inha University, Incheon, Republic of Korea; 3 WCSL of Integrated Human Airway-on-a-Chip, Inha University, Incheon, Republic of Korea; Cedars-Sinai Medical Center, UNITED STATES

## Abstract

When living tissues are injured, they undergo a sequential process of homeostasis, inflammation, proliferation and maturation, which is called wound healing. The working mechanism of wound healing has not been wholly understood due to its complex environments with various mechanical and chemical factors. In this study, we propose a novel *in vitro* wound healing model using a microfluidic system that can manipulate the topography of the wound bed. The topography of the extracellular matrix (ECM) in the wound bed is one of the most important mechanical properties for rapid and effective wound healing. We focused our work on the topographical factor which is one of crucial mechanical cues in wound healing process by using various nano-patterns on the cell attachment surface. First, we analyzed the cell morphology and dynamic cellular behaviors of NIH-3T3 fibroblasts on the nano-patterned surface. Their morphology and dynamic behaviors were investigated for relevance with regard to the recovery function. Second, we developed a highly reproducible and inexpensive research platform for wound formation and the wound healing process by combining the nano-patterned surface and a microfluidic channel. The effect of topography on wound recovery performance was analyzed. This *in vitro* wound healing research platform will provide well-controlled topographic cue of wound bed and contribute to the study on the fundamental mechanism of wound healing.

## Introduction

When wounded, the tissues of living organs work to restore the original tissue form or function through a specific physiological process called wound healing. The wound healing process is commonly encountered with skin incisions and burns. For organs such as muscle, liver, lung and kidney, soft tissue remodeling also occurs in a similar or tissue-specific process [1].

The largest organ in the human body is the skin. In this organ, the tissue recovery process is typically distinguished by a four-stage wound healing process: hemostasis, inflammation, proliferation and remodeling (or maturation) [2]. In essence, wound healing in adulthood cannot fully recover the original tissue structures and functions, unlike embryonic wound healing. For example, if a sweat gland and hair follicle are removed by the wound, then they cannot be completely reproduced, even if skin is subject to normal wound healing [2]. In severe cases, pathological wound healing induces scarring and chronic wounds, and these morbidities result in not only somatic problems but also psychological problems, decreasing the person’s quality of life due to stigmatization [3–5].

Various studies on the wound healing mechanism and treatment protocols have been conducted to improve the characteristics of skin wound healing and related diseases. Various cells (fibroblasts, endothelial cells and macrophages) and factors, such as cytokines (transformation growth factor-β1 (TGF-β1), tumor necrosis factor-α (TNF-α) and interleukin-1 (IL-1)), participate in normal and pathological wound healing and are related to each other through complex signaling pathways [6]. Fibroblasts are one of the major components in tissue remodeling and pathological healing processes [1]. Fibroblasts carry out the functions of extracellular matrix (ECM) synthesis, ECM degradation and wound contraction. Therefore, fibroblasts play an important role in wound closure and tissue remodeling. Fibroblasts migrate into the wound bed for regeneration in the proliferation phase of the early stage of wound healing. Complex signaling pathways are also involved in the mechanical and chemical cues for the wound healing process [7]. Various mechanical factors, such as tension, stiffness and topography, directly affect the mechano-responsive receptors or indirectly affect the release of biochemical factors [8–10]. The topography of substratum is known as one of major factors to make a decision of cell migration and differentiation [11]. The surface of ECM or neighboring cells are recognized as the substratum for *in vivo* cell migration in the wound healing process [12]. In particular, the topographic characteristics of ECM contribute to the migration of various cell populations in skin tissue [13] and the wound bed includes the various ECM of fibrin clot and granulation tissue [14].

Various models have been proposed to study the interactions between the wound healing process and the mechanical environment. Animal models have been widely used due to its advantages of comprehensive and systemic experiments [15, 16], while they require specialized instruments and workforces to conduct wound healing study with well-controlled experimental environments [17]. *In vitro* wound healing models with a simple design, repeatability and low cost have been proposed to resolve these issues [18, 19]. For *in vitro* wound formation methods, mechanical, electrical and optical wound formation have been studied to reproduce various types of wounds, *e*.*g*., incisions and burns [20]. For example, scratch methods have been widely used to reproduce mechanical damage [20], according to which the effects of topography on the wound healing process have been studied [21]. However, these methods interfere with heterogeneous experimental factors such as various cytokines and cell debris. Other researchers used PDMS barrier [22] and stencil [23] methods, which do not mechanically damage the cell culture region. A PDMS block is installed as a barrier and removed after sufficient cells are cultured to mimic wound generation. The empty area in which no cells exist is considered the wound. This method was used to investigate topographic effects on wound healing [24]. However, these methods can reproduce only a limited range of mechanical environments with regard to actual wounds. In particular, the cell-cell interactions of a wound bed and the associated mechanical environment are reproduced in a limited manner [25].

To overcome the shortcomings of these *in vitro* models, a chemically induced wound model was proposed using microfluidic devices [26]. Multiple fluid flows can be manipulated in spatial and temporal manners due to the low Reynolds number in microfluidic channels. Due to this flow characteristic, it is possible to dissociate cells within a selected region using a specific cell detaching solution, such as trypsin/EDTA, to form a wound-like cell-free area. This approach provides a precisely controlled experimental environment and a reproducible wound biology research platform.

In this study, we propose a novel *in vitro* wound healing model based on a nano-pattern integrated microfluidic device to study the effects of topography on the wound healing process. The nano-patterned surface recapitulates the *in vivo* fibrous ECM wound bed structure, and the microfluidic device generates wounds through fluid flow control and then reproduces the wound healing process. We cultured NIH-3T3 fibroblasts in the microfluidic device and analyzed their cellular behavior during wound healing while varying the topographical conditions. The effects of topography on wound recovery performance were investigated through the control of the nano-patterns. This *in vitro* wound healing model suggests controllable and reproducible wound environments as a research platform suitable for further experiments and contributes to the basic study of the mechanisms of wound biology.

## Material and methods

### Fabrication of nano-patterns and a microfluidic device

Rectangular-shaped nano-patterns (S1 Fig) were fabricated based on PDMS (polydimethylsiloxane) replication with a polyurethane-acrylate (PUA) mold generated by capillary force lithography (provided by Prof. Kahp Yang Suh, Seoul National University, South Korea). PDMS elastomer and curing agent (Sylgard 184 Dow Corning, USA) were mixed at a 10:1 weight ratio, and the mixture was poured onto the PUA mold to replicate the nano-patterns with PDMS. Then, the PDMS on the PUA mold was baked for 4 hours at 65°C in a convection oven (NDO-400, Eyera, Japan) to achieve polymerization. After baking, a cured PDMS slab, which included replicated nano-patterns, was detached from the PUA mold. The dimensions of the nano-patterns were investigated by scanning electron microscopy (SNE-3200M, SEC, South Korea) (S1 Fig). The height of the nano-pattern was ~320 nm, the width of the ridge was 550 nm and the width of the groove was 550, 1100 or 2750 nm. The ratio of the width of the ridges to grooves was 1:1, 1:2 or 1:5, respectively.

**Fig 1:**
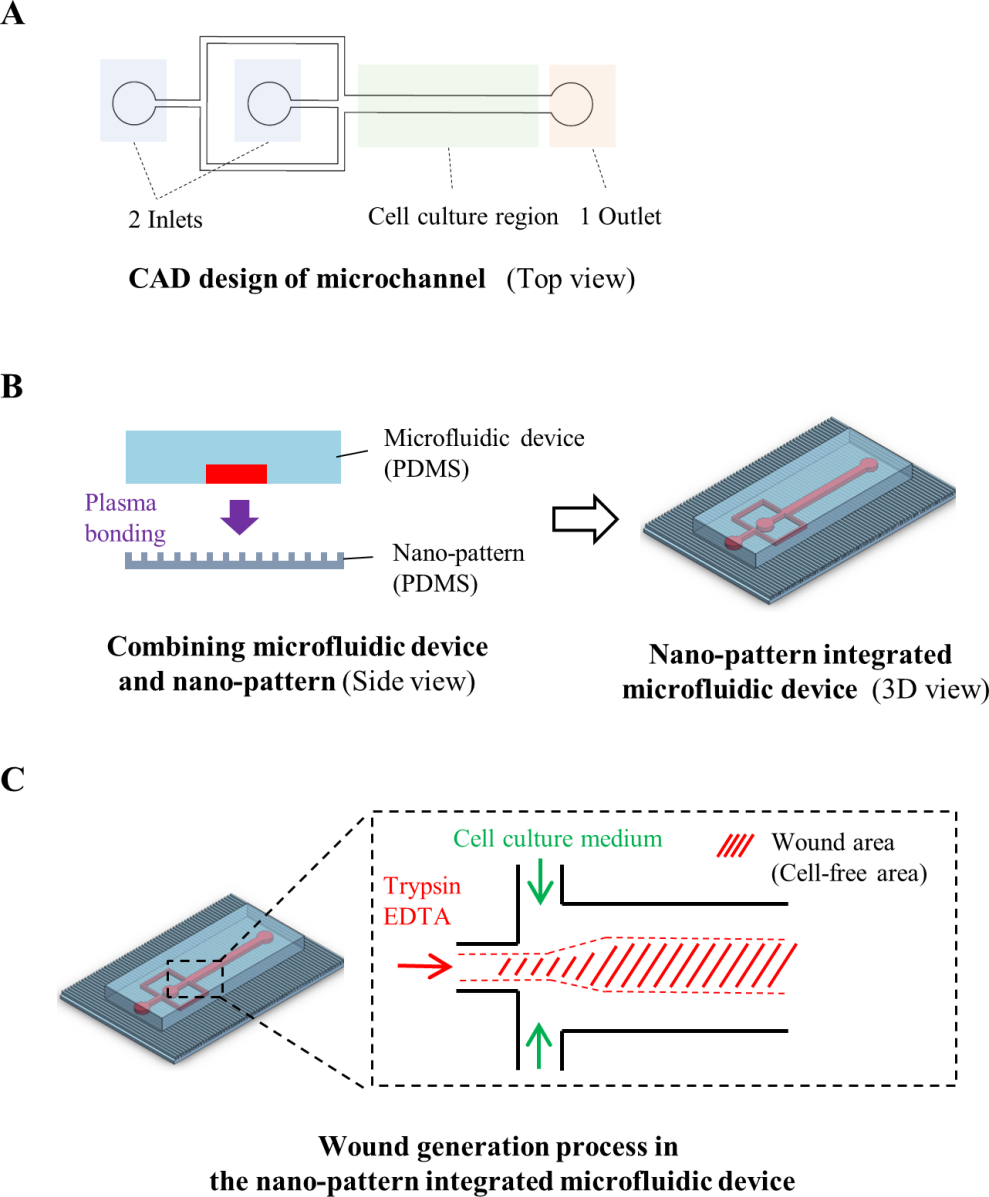
Nano-pattern integrated microfluidic system used to reproduce the wound healing process. **(A)** CAD design of the microfluidic device, which includes 2 inlets, 1 outlet and a cell culture region. **(B)** Process used to fabricate the microfluidic channel and nano-pattern. The device and nano-patterns were irreversibly combined through plasma bonding methods. **(C)** 3D image of the combined microfluidic device with the nano-pattern. Schematic of the *in vitro* wound formation process in the microfluidic channel using the layered flow of trypsin/EDTA. Due to trypsinization, cells detached from the microfluidic channel, allowing the selective formation of a cell-free area.

A microfluidic device for a wound generation/healing assay was designed using CAD software (AutoCAD, Autodesk, USA). The microfluidic device had two inlets and one outlet, and the cell culture region of the device was 0.5 mm wide, 10 mm long and 0.2 mm high (Fig 1A). A mold of the microfluidic device was fabricated by soft lithography using SU-8 photoresist (SU-8 2050, Microchem, USA) [27]. PDMS microchannels were replicated from an SU-8 mold by using the same nano-pattern fabrication process. A replicated PDMS microfluidic channel was bonded to a nano-patterned PDMS slab after air plasma treatment (CUTE, Femto Science, South Korea) (Fig 1B). The bonded device was baked at 65°C overnight in a convection oven to enhance bonding. Two inlets and one outlet port were punched for cell loading and media supply using a biopsy punch (Miltex®, Ted Pella, USA).

### Cell culture and harvesting

Mouse embryo origin NIH-3T3 fibroblasts [28] (21658, SNU cell bank, South Korea) were cultured in a 75 ml cell culture flask (Corning, USA) with 10% fetal bovine serum (FBS) (10082147, Gibco, USA) and 1% penicillin/streptomycin (15140122, Invitrogen, USA) in Dulbecco’s Modified Eagle Medium (DMEM) (11965092, Invitrogen, USA) [29]. Then, the cell culture flask was incubated in a 5% CO_2_ and 37°C incubator (HeraCell 150, Thermo Scientific, USA) for 2 to 3 days until the cell population exceeded 90% of the flask. Cells were harvested through trypsinization using 0.05% trypsin-EDTA (25200056, Gibco, USA) and then diluted to a specific concentration and seeded onto the nano-patterned surface in a microfluidic device.

### Cell elongation and orientation analysis

The surface of the nano-patterned PDMS was modified by an oxygen plasma generator to form a hydrophilic layer for cell culture. Then, the nano-patterned PDMS was washed using 1x Dulbecco's Phosphate-Buffered Saline (DPBS) (14190–144, Invitrogen, USA) and sterilized using a UV lamp (UV-LF2016-LS, UVItec, England) for 10 min. 40μg/ml fibronectin solution (F1141, Sigma Aldrich, USA) solution was deposited onto the nano-patterned surface of the to promote cell adhesion and then incubated overnight at 5% CO_2_ and 37°C. The harvested NIH-3T3 fibroblasts were seeded onto the nano-patterned surface at a density of 0.5×10^6^ cells/ml and were incubated at 5% CO_2_ and 37°C. After cell seeding, microscopy images were captured using an inverted microscope (Eclipse Ti-U, Nikon, Japan) every 2 hours for 6 hours. ImageJ software (NIH, USA) was used to analyze the effects of the surface topography on the cell morphology, specifically the elongation and orientation from captured images. Cell elongation and orientation were analyzed through the following indices;

$$\mathrm{Elongation}\;\mathrm{index},E=\frac{\mathrm{Cell}\;\mathrm{body}\;\mathrm{length}\;\mathrm{on}\;\mathrm{long}\;\mathrm{axis}}{\mathrm{Cell}\;\mathrm{body}\;\mathrm{length}\;\mathrm{on}\;\mathrm{short}\;\mathrm{axis}}$$

Orientationangle,θ=Anglefromnano‑patterntolongaxisofcellbody

### Fluorescence assay

Four percent paraformaldehyde (P6148, Sigma Aldrich, USA) in 1x DPBS and 0.5% of Triton-X solution (T8787, Sigma Aldrich, USA) in 1x DPBS were used for cell fixation and permeabilization. Then, the nuclei and F-actin of the cells were stained using a solution of 2 μg/ml DAPI (D9542, Sigma Aldrich, USA) and 100 mg/ml Phalloidin (P1951, Sigma Aldrich, USA) that was mixed for 2 hours at room temperature. ProLong® Gold Antifade Reagent (P36930, Invitrogen, USA) was applied to prevent fluorescence bleaching.

### Cell migration assay

The microfluidic device was washed with 1x DPBS and sterilized with a UV lamp for 10 min. Then, a 4% fibronectin solution was introduced to the microfluidic channel, and the solution was incubated at 37°C and 5% CO_2_ overnight. NIH-3T3 fibroblasts were seeded at 0.5 × 10^6^ cells/ml in the device and then incubated. During incubation, microscopy images were captured every 2 hours for 6 hours. Cell migration, specifically, migrating distance and orientation, were analyzed using ImageJ software.

### Wound generation/healing process in a microfluidic device

NIH-3T3 fibroblasts (5–6 × 10^6^ cells/ml) were cultured in the device using the same procedure as that for the cell migration assay, and cell culture medium was supplied through the inlets for 1 to 2 days until the cells were fully confluent in the device. For wound formation, we replaced the culture medium with trypsin/EDTA for 1 of the 2 inlet reservoirs. Then, we applied negative pressure using a syringe pump from the outlet port at a flow rate of 5 to 15 μl/min to create a laminar flow of trypsin/EDTA at the center (Fig 1C). After 10 to 15 min, a cell-free area clearly formed where only the trypsin/EDTA treatment had been applied. Finally, trypsin/EDTA was replaced with normal DMEM, and the device was returned to the incubator to observe the wound healing phase.

### Recovery index analysis

The recovery index was defined to quantitatively analyze the effects of topography on the wound healing process:

$$\mathrm{Recovery}\;\mathrm{index}=\frac{\mathrm{Cell}‑\mathrm{free}\;\mathrm{area}}{\mathrm{Initial}\;\mathrm{cell}‑\mathrm{free}\;\mathrm{area}}$$

The cell-free area is identified as the wounded region in the cell cultured microchannel formed by the wound generation process. Images were captured using a microscope every 2 hours for 20 hours, and the cell-free area was measured using ImageJ software. The initial cell-free area was measured immediately after wound generation.

## Results

### Cell shape analysis on the nano-patterns

**Fig 2:**
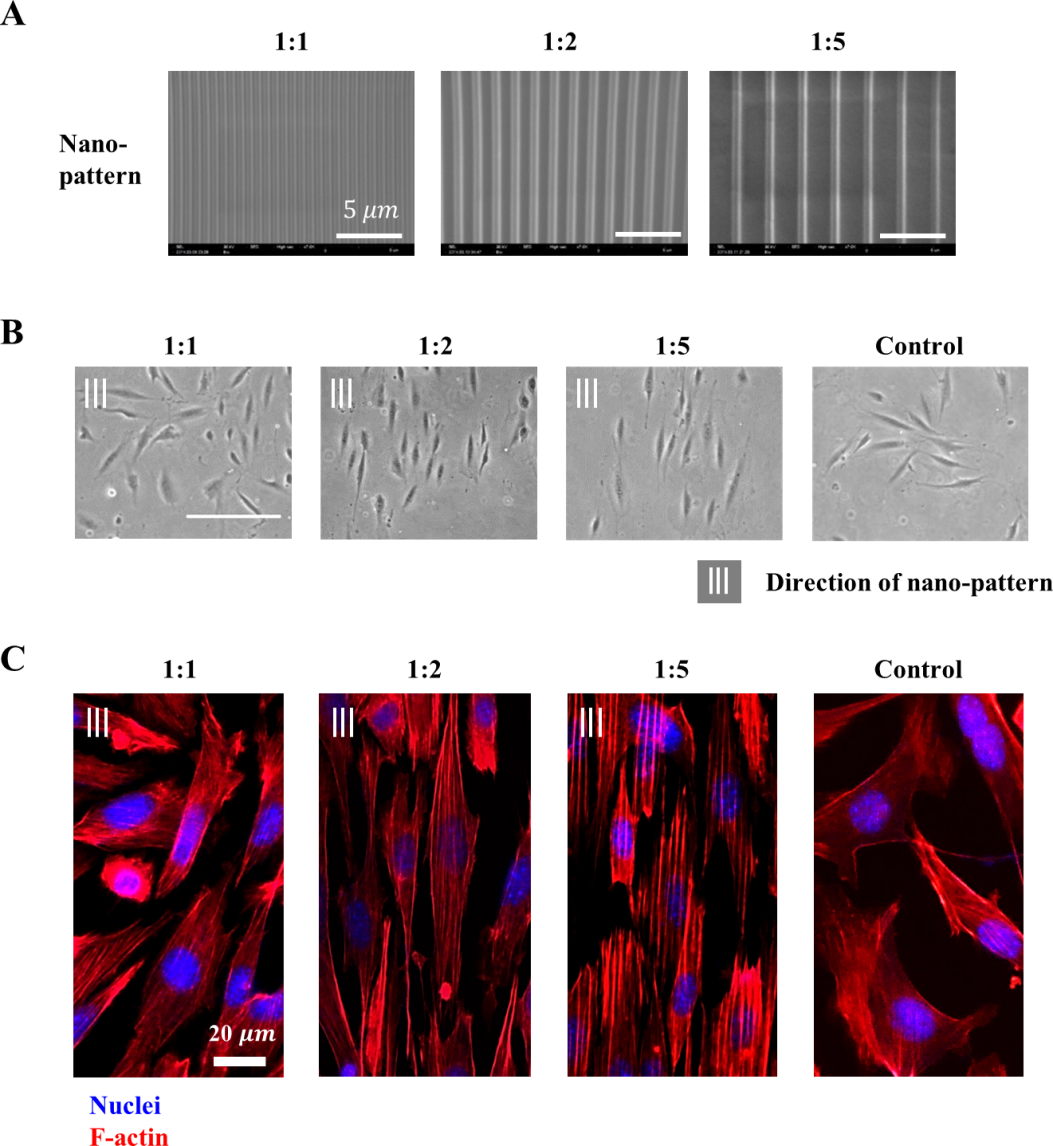
Cell culture on various nano-pattern densities. **(A)** SEM images of the nano-patterns (1:1, 1:2 and 1:5). Dimensions of the nanostructure were measured as width 560 ± 20 nm and depth 325 ± 3 nm. The gap between the structures varies for each case. A gap equal to the width of the nanostructure was considered the 1:1 ratio nano-pattern. Further, we constructed 1:2 and 1:5 ratio nano-patterns in which the dimension of the gap was 2 or 5 times broader than the width of the ridge. **(B)** Microscopic images of NIH-3T3 fibroblasts cultured on the nano-pattern (cell density = 300–400 cells/mm^2^, scale bar = 100 μm). **(C)** Fluorescence images of stained cells on nano-patterned surface using nuclei (DAPI) and F-actin (Phalloidin).

NIH-3T3 fibroblasts were cultured on nano-patterns to analyze the effect of nano-patterns on cell behavior. First, the cell body shape was observed on the nano-pattern. The nano-patterns contained unidirectionally aligned ridges and grooves, and each device had one of three ratios of ridge to groove width: 1:1, 1:2 or 1:5 (Fig 2A). The morphology of the cultured cells was observed using an inverted microscope for 12 hours after cell seeding (Fig 2B). Then, a fluorescence assay was applied to observe the alterations in the intracellular environments, specifically the protein level (Fig 2C). Cell nuclei (blue) and F-actin (red), one of major components of the cytoskeleton that supports the cell body, were stained.

**Fig 3:**
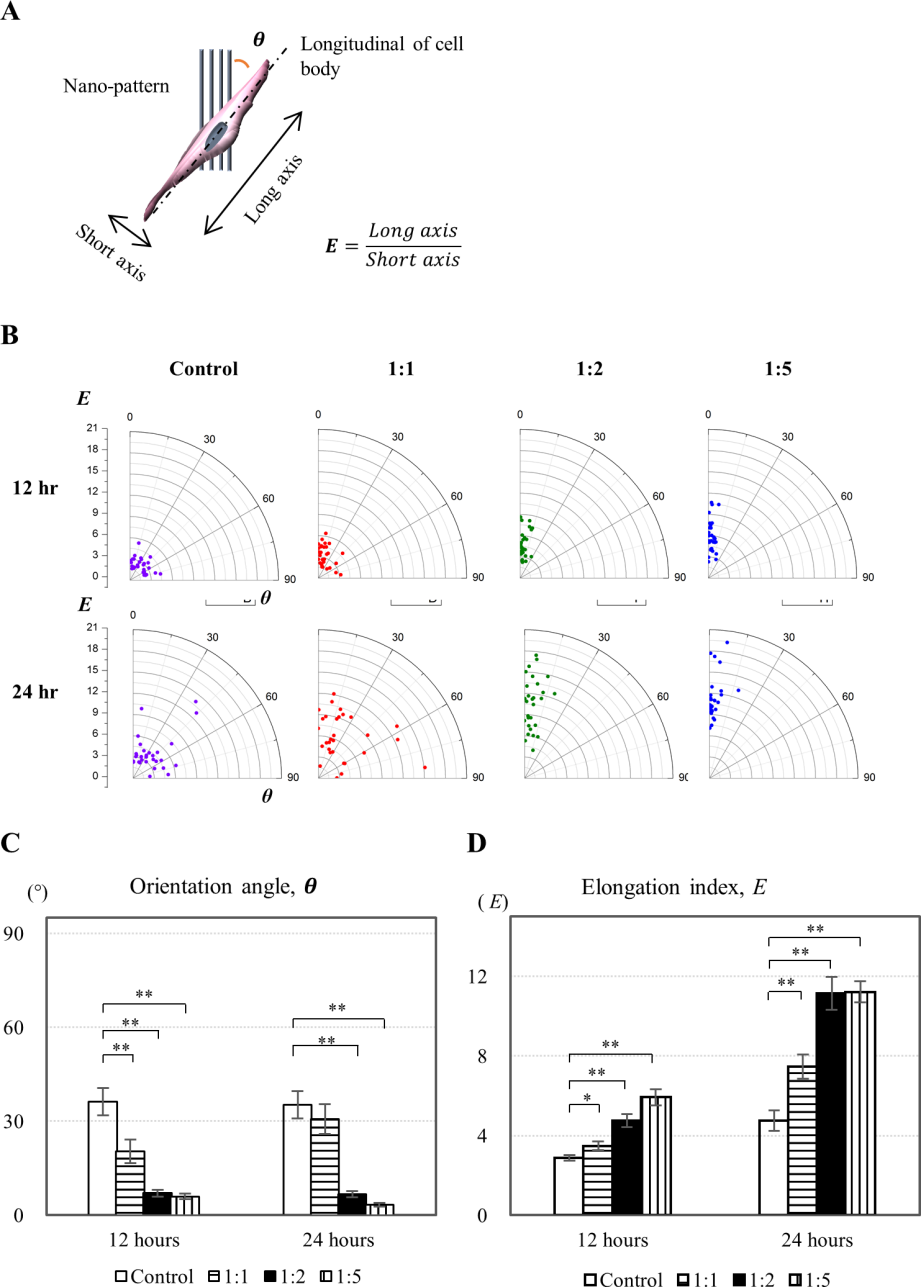
Orientation and elongation of cells on the nano-patterns. NIH-3T3 fibroblasts on nano-patterns were investigated based on their elongation and orientation following two indices to validate the contact guidance effect from various densities of nano-patterned surfaces. **(A)** Definition of the indices for cell body orientation and elongation. Orientation angle, *θ*, is the angle between the nano-pattern and longitudinal length of the cell body. Elongation index, *E*, is the aspect ratio of the long axis to the short axis of the cell body. **(B)** Orientation angle and elongation index of cells on the various nano-patterns: 1:1, 1:2 and 1:5 nano-pattern ratios and control (no-pattern). Cellular shape was captured using a microscope at 12 and 24 hours after cell seeding. Average values of **(C)** orientation angle, *θ*, and **(D)** elongation index, *E* (n = 30, *p < 0.05 and **p < 0.01).

We defined two indices, an orientation index, *θ*, and an elongation index, *E*, to quantitatively analyze the morphological changes of the NIH-3T3 fibroblasts on the nano-pattern (Fig 3A). The indices *θ* and *E* were measured at 12 and 24 hours after cell seeding (Fig 3B).

When the orientation index *θ* was near 0°, the cells were significantly more aligned with the direction of the nano-pattern. As shown in Fig 3B, the 1:2 and 1:5 ratio patterns showed *θ* close to 0°, whereas the cells were more closely aligned with the nano-pattern at the 1:1 ratio. In contrast, the control case presented radially distributed *θ* values, with cells that were randomly stretched in various directions. Fig 3C clearly shows that the cells were statistically (Student’s t-test, N = 30, **p < 0.01) more aligned on the 1:2 and 1:5 ratio patterns.

For the elongation index *E* in Fig 3B, the 1:2 and 1:5 ratio patterns showed a higher *E* value than the 1:1 ratio pattern and control cases, and the cell body was more extended or stretched on the 1:2 and 1:5 ratio patterns. *E* values increased with respect to time due to cell proliferation. Fig 3D statistically proves these spatial and temporal elongation trends (Student’s t-test, N = 30, *p < 0.05, **p < 0.01).

### Cell migration on the nano-patterns

**Fig 4:**
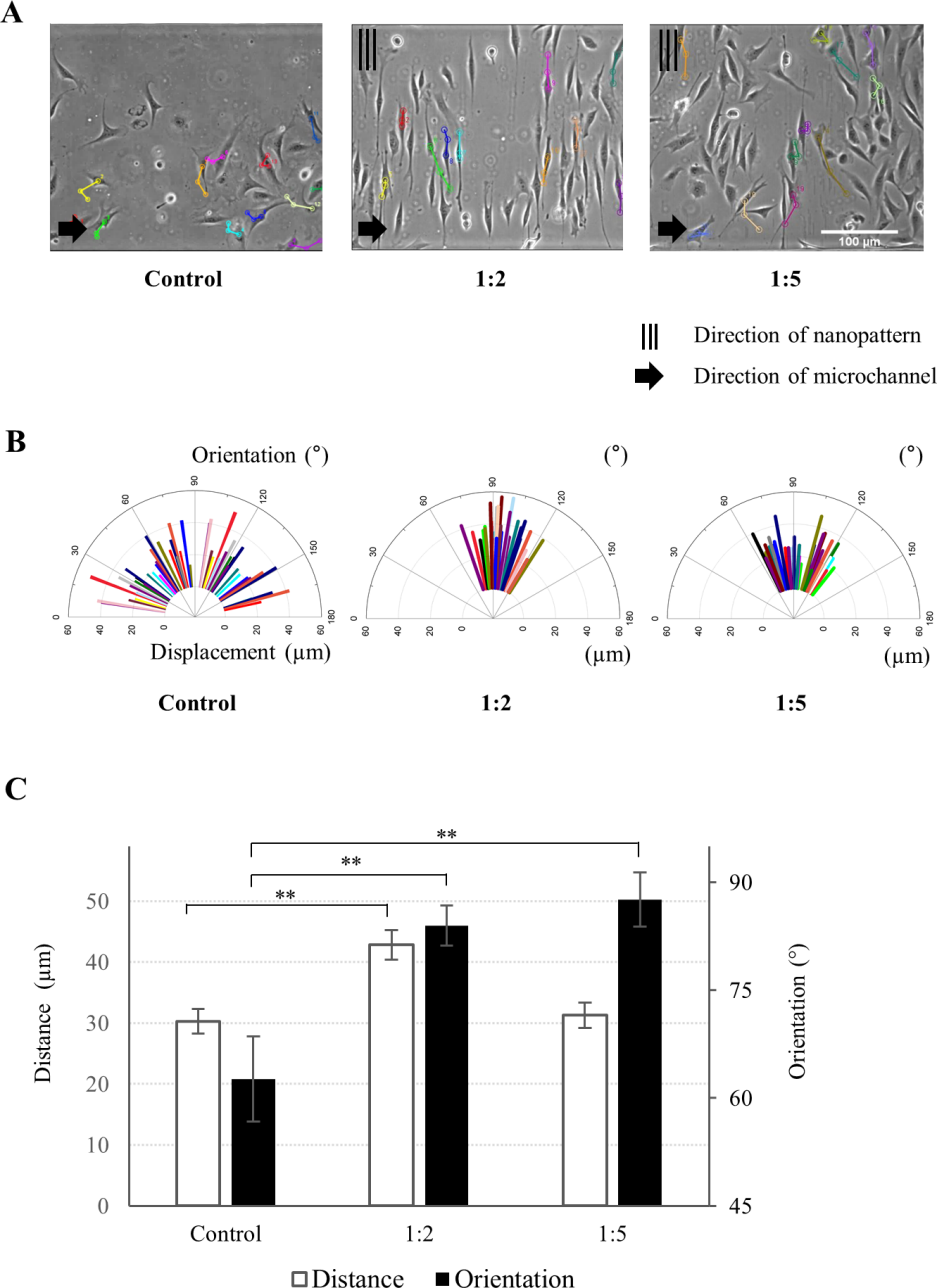
Cell migration on nano-patterned surfaces. **(A)** Micrographs of NIH-3T3 fibroblast migration on the nano-patterned surfaces. Colored lines indicate the distance and orientation of cell migration. After cell seeding, cell movements were captured every 2 hours for 6 hours. **(B)** Distribution of cell migration orientation and distance (n = 39). Orientation angle was the averaged value, and distance was summed over 3 measurement points over a total of 6 hours. **(C)** Average values of the migrated distance and orientation of cells cultured on nano-patterns and the control surface (n = 39, **p < 0.01).

Cell migration is an important feature of the wound healing process. NIH-3T3 fibroblasts were cultured in the nano-pattern integrated microfluidic device to analyze the effect of the nano-pattern on cell migration. The 1:2 and 1:5 ratios of the nano-patterns were integrated with the microchannel because these ratios of patterns presented relatively high cell elongation and alignment as presented in the previous section. NIH-3T3 fibroblasts cultured in the device were monitored using microscopy for 6 hours at intervals of 2 hours after cell seeding. The migrating distance and orientation of the cells were measured using the cell tracking plugin of ImageJ (Fig 4A). The migrating distance was calculated based on the summation of the distance during the time interval of 6 hours. The nano-pattern was aligned perpendicular to the microfluidic channel, and the migrating orientation was measured from the channel direction. For example, if the cell movement was parallel to the microfluidic channel, then the orientation was 0°. Fig 4B shows the migration patterns of cells on the control, 1:2 and 1:5 ratio nano-patterns. The average cell migration distance on the 1:2 ratio nano-pattern was 42.83 μm, which was longer than that on the 1:5 ratio nano-pattern (30.33 μm) and the control surface (31.28 μm). The average migration orientations on the 1:2 and 1:5 ratio nano-patterns were similar (Fig 4C). In contrast, the cells on the control surface migrated in arbitrary directions with no specific trend.

### *In vitro* wound generation/healing in the microfluidic device

**Fig 5:**
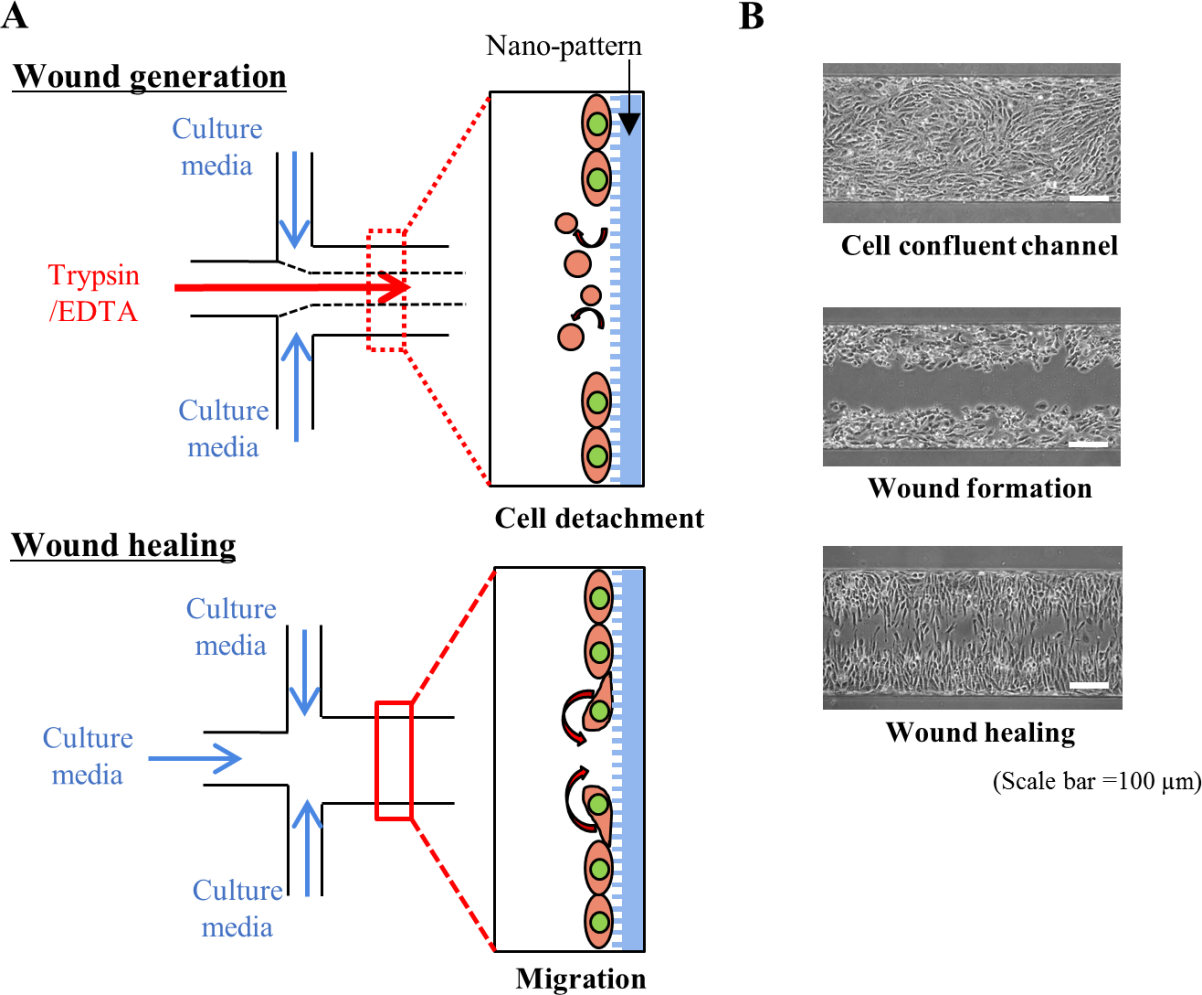
Wound generation and healing processes in the microfluidic devices. **(A)** Schematic of wound formation and healing processes in a nano-pattern integrated microfluidic device. First, the wound is formed due to the layered flow of trypsin/EDTA. The enzyme induces cell detachment from the nano-patterned surface within a selective region. Trypsin/EDTA is replaced with culture medium after wound formation is finished. Leading cells on the wound edge recover into the cell-free area (wound healing process). **(B)** Micrographs of the wound formation and healing processes. Sequentially, from the top: the cell culture, wound formation and wound healing stages.

During the wound generation process, a layered flow of trypsin/EDTA was formed at the center of the microchannel by the flow configuration shown in Fig 5A. We applied a flow rate of 5 to 15 μL/min to form a 250 to 300 μm-wide cell-free area, which constituted the wound (S2 Fig). After the cell-free area had been generated, trypsin/EDTA was replaced with DMEM again, and the device was incubated to represent the wound healing phase, in which cells recover into the cell-free area (Fig 5B).

### Effects of the nano-pattern on the wound healing process

**Fig 6:**
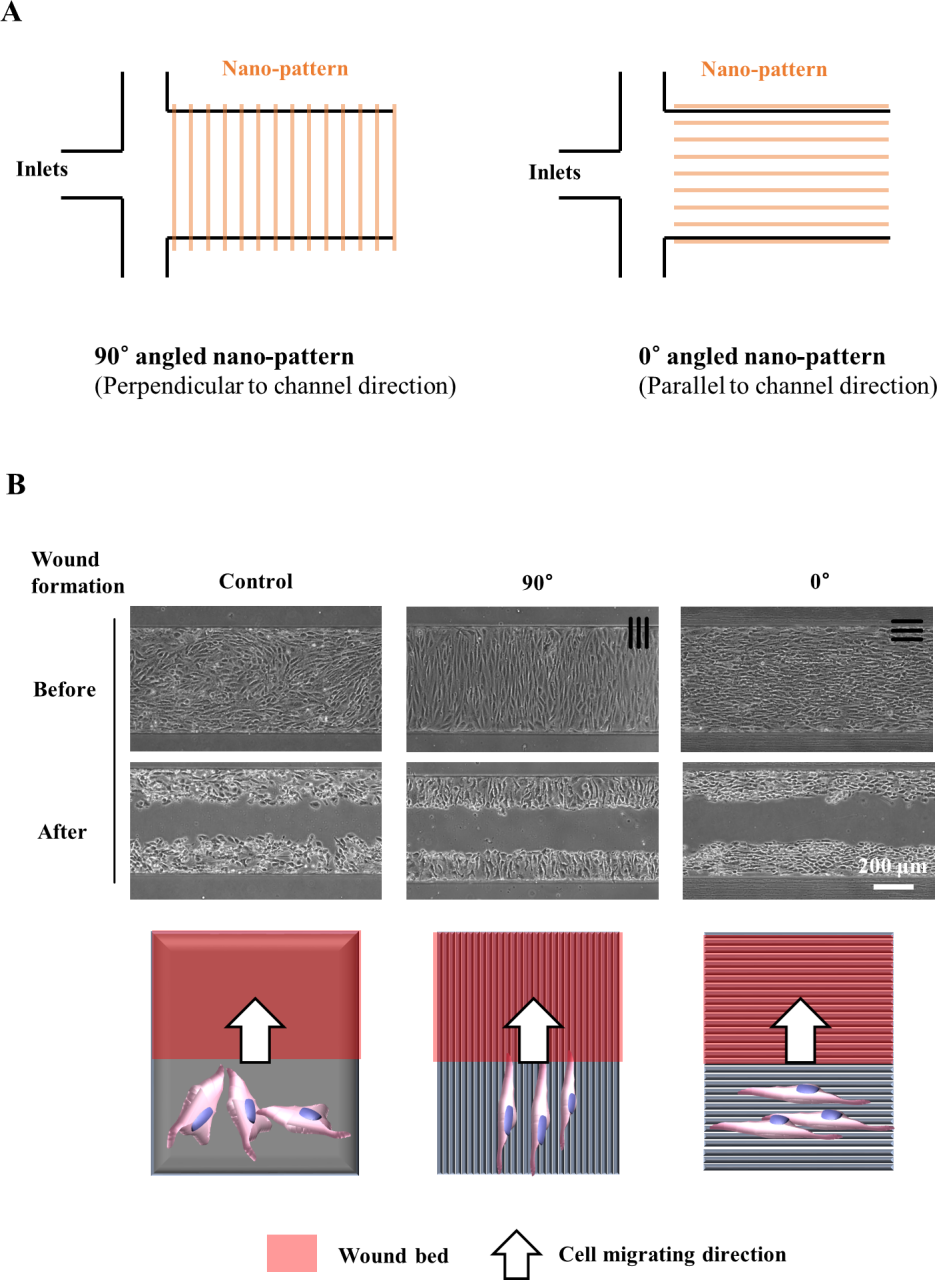
Nano-pattern integrated microfluidics were used to represent different topographical environments for NIH-3T3 fibroblast in the wound healing process. **(A)** A nano-pattern was integrated with a microfluidic device to present different topographical environments in the wound healing process. The nano-pattern was either perpendicular or parallel to the channel direction. **(B)** Confluent NIH-3T3 fibroblasts in the microfluidic channel, which includes the nano-pattern and schematics of the wound healing process after wound formation.

The aligned angle of the nano-pattern with the microchannel was manipulated to investigate its effects on the wound recovery rate. The nano-pattern was aligned either parallel (0°) or perpendicular (90°) to the microchannel (Fig 6A). After the wound formation process, the cells on the wound edge faced different nano-patterned structures (Fig 6B). The 1:2 ratio nano-pattern was selected for integration with the microchannel because the 1:2 ratio nano-pattern showed a comparably high contact guidance performance and fast cell migration to those the other ratios of nano-patterns.

**Fig 7:**
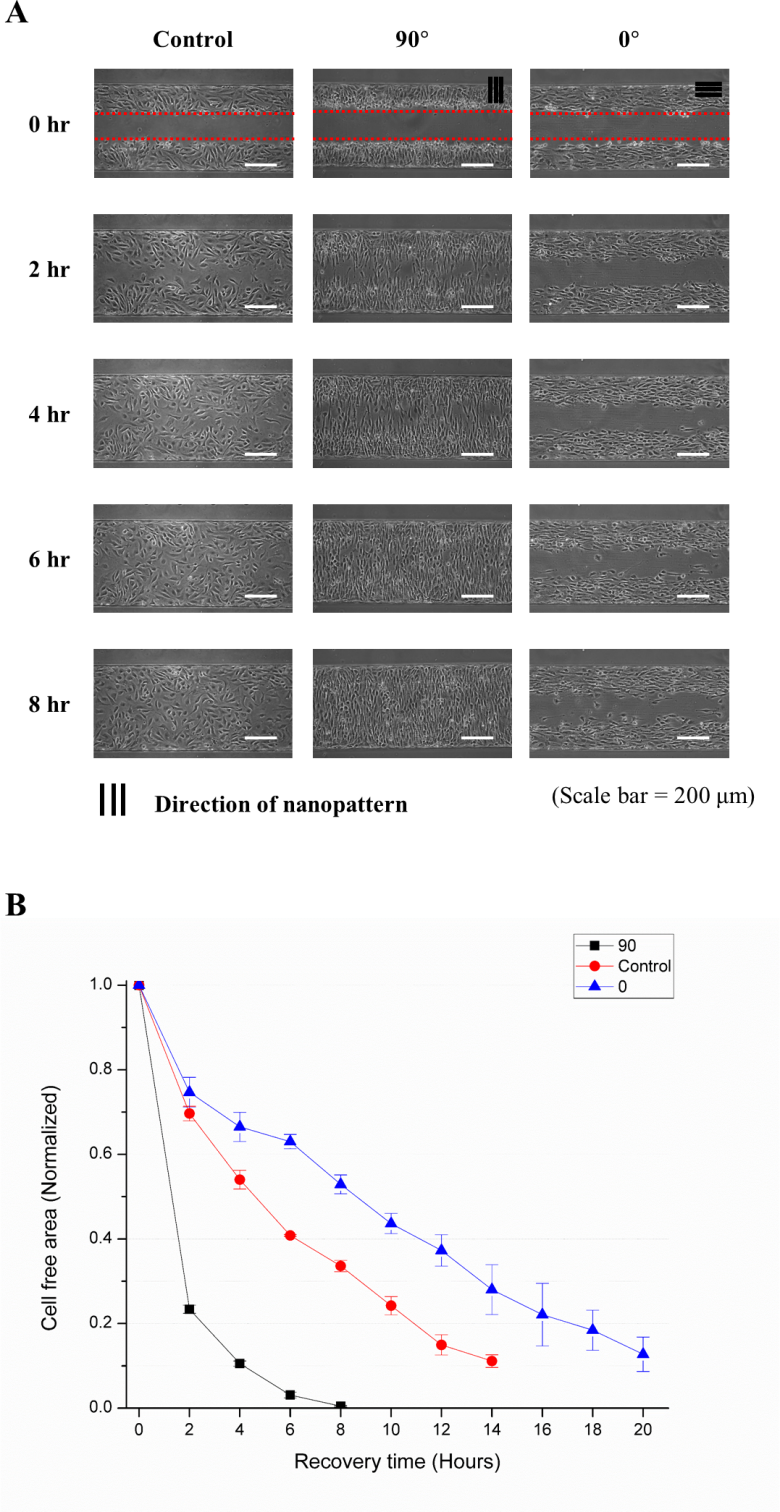
*In vitro* wound healing process and recovery rate. **(A)** Time-lapse images of the wound healing process on the device (every 2 hours for 8 hours). **(B)** Recovery index of different nano-patterns and the control surface. The 90° nano-pattern showed the fastest recovery rate. However, the 0° nano-pattern showed an even slower recovery rate than the control surface.

The wound healing phase was monitored every 2 hours after wound formation until the cell-free area had been mostly filled by cells (Fig 7A). Fig 7B shows the recovery indices of each case during wound recovery, where 90° alignment showed the fastest recovery, as expected.

## Discussion

Both cell morphology and dynamic cellular behaviors were modified by the nano-patterns and the effects of nano-patterns were exhibited in a recapitulated wound healing process in the microfluidic system.

First, the static behaviors of the NIH-3T3 fibroblasts cultured on the nano-patterned surface exhibited an extended and aligned body shape along the nano-pattern. There were differences in the degree of extension and alignment based on the ratio of the nano-pattern. Specifically, the 1:2 and 1:5 ratio nano-patterns showed a significantly higher degree of cell body extension and alignment along the nano-pattern than the 1:1 ratio nano-pattern and control. Fluorescence images of the intracellular structure supported these results at the protein level. F-actin was stained to observe the deformation of intracellular structure by the nano-patterns because the remodeled cytoskeleton that is linked to the adhesion molecules is one of the key evidences for contact guidance. Specifically, the F-actin network and nuclei were aligned with the nano-pattern but an arbitrarily formed F-actin network was observed on the control surface.

It is clear that the specific threshold ratio of the nano-pattern can induce contact guidance, thus controlling cell body orientation and its extension based on geometry. The topography of substratum induces contact guidance through regulating the adhesion molecules of cells [30]. Lamellipodia on the nano-patterns are regulated to express adhesion molecules along with the nano-patterns, resulting in directed cell adhesion and migration processes [31]. Saito, A.C. et al. studied signaling pathways with regards to adhesion molecules in contact guidance on the nano-patterns [32]. In detail, the authors revealed that the groove of the nano-patterns regulates focal adhesion protein, such as paxillin, integrin, expression and the maturation of adhered cells. In our study, a specific ratio of the nano-patterns between 1:1 and 1:2 is expected to regulate the maturation of focal adhesion protein and induce contact guidance.

Second, the dynamic behaviors of the cells were controlled by the nano-patterns. Migration analysis (Fig 4B and 4C) indicated that the direction of cell movement was nearly consistent with the nano-pattern orientation (90°). In the control experiment, cells were widely spread throughout the angles ranging from 0 to 180°. Previous studies showed that the spatial confinement of focal adhesion (FA) proteins guides cell migration since cells form FAs to be adhered to substrates [33, 34]. On the anisotropic nano-patterns, FAs were spatially confined within the ridges and linked with cytoskeletons, exerting contractile forces for translocation [35, 36]. As a result, cell protrusion and migration follow the same direction along the nano-pattern. Therefore, NIH-3T3 fibroblasts on the 1:2 and 1:5 ratio nano-patterns also presented anisotropic migration.

Based on an analysis of cellular behaviors on the nano-patterns, we confirmed that this nano-pattern integrated microfluidic device can represent an *in vitro* wound healing process. Flow in the nano-pattern integrated microfluidic device was controlled to represent a wound healing process. In particular, it was possible to form a layered flow, that is, a spatially separated flow, in the microchannel.

A cell-free area was generated by introducing trypsin/EDTA flow at the center region of the microchannel. Two primary mechanisms led to this cell-free area. First, due to the low Reynolds number in the microfluidic channel, it was possible to form trypsin/EDTA flow at a specific region, that is, at the center region with side sheath flows. Second, the system had a high Peclet number, *Pe*, which is a dimensionless number defined as the ‘advective transport rate’ over the ‘diffusive transport rate’ in the trypsinization process [25]. This number determines the system’s tendency toward convection or diffusion. In the microchannel, convection was dominant over diffusion due to the relatively low diffusivity of trypsin (D_*trypsin*_ = 2.1 × 10^−10^ m^2^/s). Even as the trypsin/EDTA in the center region diffused to both sides, fetal bovine serum (FBS) in the side flows of the cell culture medium inhibited trypsinization by protease inhibitors, such as α1-antitrypsin and α2-macroglobulin. Therefore, we were able to develop a cell-free area in a controlled manner in our system.

The effects of topography on the wound healing process were investigated using a nano-pattern integrated microfluidic system. Specifically, the 1:2 ratio nano-pattern was used because this pattern induced the longest migrating distance of the NIH-3T3 fibroblasts. The nano-pattern perpendicular to the microchannel (90° case) showed a recovery index of almost 0 at 8 hours, which means that this alignment showed the fastest recovery rate among all cases. This result showed that cell motility was affected by the nano-pattern, even during collective migration, and that the recovery rate in general can be accelerated by topography control. It should be noted that parallel alignment (0° case) showed a slower recovery rate than the flat surface (control). This tendency is consistent with previous research that focal adhesion protein formation ‘across ridge to ridge’ is disadvantageous compared to that ‘along the ridge’ from the perspective of mechanical tension [32]. These results suggest that cell migration may be restricted by nanoscale geometry, which could be applied to achieve excessive activation of NIH-3T3 fibroblasts in fibrosis-related diseases.

## Conclusion

In this study, we utilized various nano-patterns in cell culture to mimic the aligned structure of collagen fiber and fibroblasts in skin dermal layer and investigated the effects on the morphology and dynamic behaviors of cells, including the orientation, elongation and migration of NIH-3T3 fibroblasts. We fabricated a nano-pattern integrated microfluidic device that recapitulated the physiological wound healing process on a chip. To reproduce wound formation, selective trypsinization was induced based on the layered flow of trypsin/EDTA in the microchannel. The wound generation and healing processes were analyzed for nano-patterns that were aligned with the microchannel. Nano-pattern alignment affected the collective cell migration trends under different geometric conditions during the wound healing process. This study has developed as a basic research model that can be used in physiological, pathological wound healing studies. The developed system can serve as a test platform for skin medication and cosmetic materials.

## Supporting information

S1 Fig Structure and SEM images of various nano-patterns. The ratio of ridge to groove of the nano-patterns for each surface (1:1, 1:2 and 1:5). SEM images show the section and top views of each nano-pattern.
(TIF)

S2 Fig Layered flow control in a microfluidic device. Flow rate was controlled at the channel outlet using a syringe pump. Distilled water (DIW) and 5% black ink diluted with DIW were each injected through one of two inlets. Different widths of layered flow were formed with respect to the flow rate.
(TIF)
