# Supplementary figures and images for: Investigation of wound healing process guided by nano-scale topographic patterns integrated within a microfluidic system
Source: PLoS One. 2018 Jul 26;13(7):e0201418. doi: 10.1371/journal.pone.0201418 (PMC6062108; doi:10.1371/journal.pone.0201418)

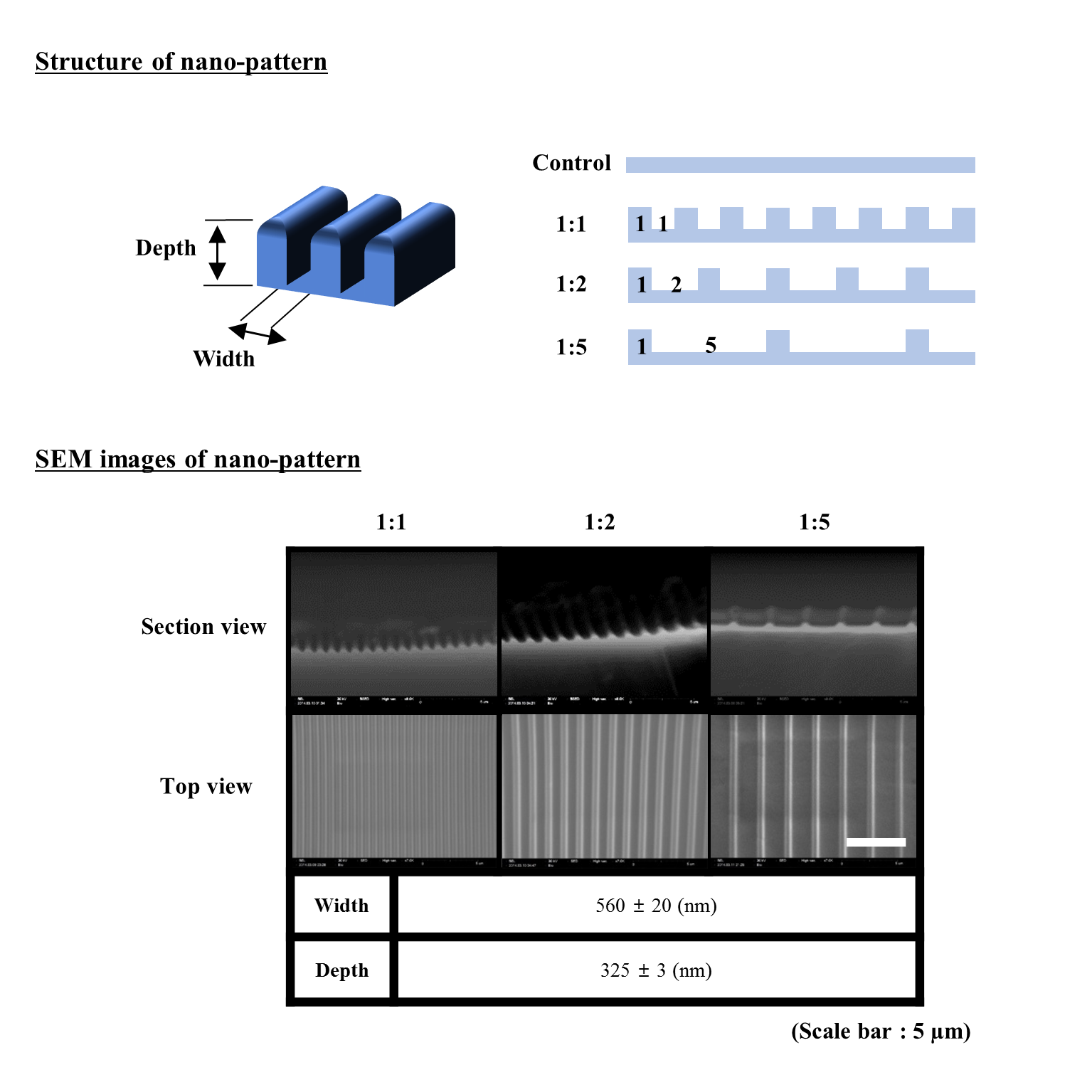

Supplement: S1 Fig — The ratio of ridge to groove of the nano-patterns for each surface (1:1, 1:2 and 1:5). SEM images show the section and top views of each nano-pattern. (TIF) [file pone.0201418.s001.tif]

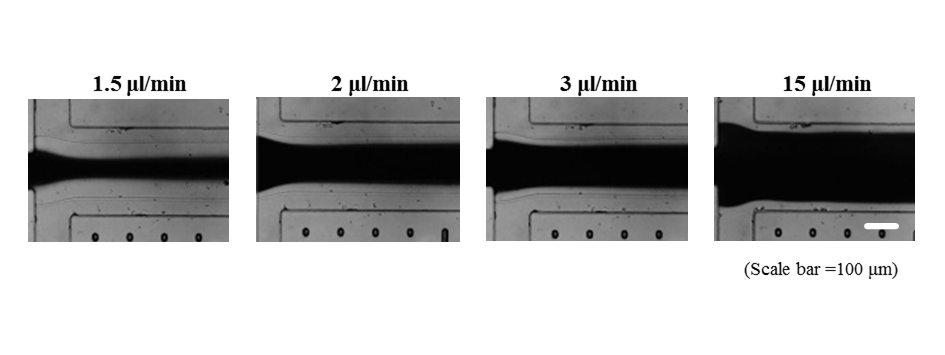

Supplement: S2 Fig — Flow rate was controlled at the channel outlet using a syringe pump. Distilled water (DIW) and 5% black ink diluted with DIW were each injected through one of two inlets. Different widths of layered flow were formed with respect to the flow rate. (TIF) [file pone.0201418.s002.tif]
